# Trends in psychiatric diagnoses by COVID-19 infection and hospitalization among patients with and without recent clinical psychiatric diagnoses in New York city from March 2020 to August 2021

**DOI:** 10.1038/s41398-022-02255-8

**Published:** 2022-11-21

**Authors:** Yunyu Xiao, Mohit M. Sharma, Rohith K. Thiruvalluru, Catherine Gimbrone, Myrna M. Weissman, Mark Olfson, Katherine M. Keyes, Jyotishman Pathak

**Affiliations:** 1 Department of Population Health Sciences, Weill Cornell Medicine, NewYork-Presbyterian, New York, NY USA; 2 Department of Epidemiology, Mailman School of Public Health, Columbia University, New York, NY USA; 3 Department of Psychiatry, Vagelos College of Physicians and Surgeons, Columbia University, New York, NY USA; 4 Division of Translational Epidemiology, New York State Psychiatric Institute, New York, NY USA; 5 Columbia University Irving Medical Center, New York, NY USA

**Keywords:** Diseases, Psychiatric disorders

## Abstract

Determining emerging trends of clinical psychiatric diagnoses among patients infected with the SARS-CoV-2 virus is important to understand post-acute sequelae of SARS-CoV-2 infection or long COVID. However, published reports accounting for pre-COVID psychiatric diagnoses have usually relied on self-report rather than clinical diagnoses. Using electronic health records (EHRs) among 2,358,318 patients from the New York City (NYC) metropolitan region, this time series study examined changes in clinical psychiatric diagnoses between March 2020 and August 2021 with month as the unit of analysis. We compared trends in patients with and without recent pre-COVID clinical psychiatric diagnoses noted in the EHRs up to 3 years before the first COVID-19 test. Patients with recent clinical psychiatric diagnoses, as compared to those without, had more subsequent anxiety disorders, mood disorders, and psychosis throughout the study period. Substance use disorders were greater between March and August 2020 among patients without any recent clinical psychiatric diagnoses than those with. COVID-19 positive patients (both hospitalized and non-hospitalized) had greater post-COVID psychiatric diagnoses than COVID-19 negative patients. Among patients with recent clinical psychiatric diagnoses, psychiatric diagnoses have decreased since January 2021, regardless of COVID-19 infection/hospitalization. However, among patients without recent clinical psychiatric diagnoses, new anxiety disorders, mood disorders, and psychosis diagnoses increased between February and August 2021 among all patients (COVID-19 positive and negative). The greatest increases were anxiety disorders (378.7%) and mood disorders (269.0%) among COVID-19 positive non-hospitalized patients. New clinical psychosis diagnoses increased by 242.5% among COVID-19 negative patients. This study is the first to delineate the impact of COVID-19 on different clinical psychiatric diagnoses by pre-COVID psychiatric diagnoses and COVID-19 infections and hospitalizations across NYC, one of the hardest-hit US cities in the early pandemic. Our findings suggest the need for tailoring treatment and policies to meet the needs of individuals with pre-COVID psychiatric diagnoses.

## Introduction

High rates of psychiatric disorders [1–3] have been reported in patients infected with severe acute respiratory syndrome coronavirus 2 (SARS-CoV-2) as a manifestation of post-acute sequelae of SARS-CoV-2, or long COVID [4–6]. Accumulating data further suggests pre-COVID psychological distress may increase the risks of long-term psychiatric illness after COVID-19 exposures [7]. However, most published report on post-COVID psychiatric illness has been based on individual self-reports of pre-COVID psychiatric history instead of clinical diagnoses [8]. Therefore, a full understanding of the long-term trends in post-COVID clinical psychiatric diagnoses is not yet known, especially regarding how trends may differ among people with and without pre-COVID psychiatric diagnoses [9–11]. It is unclear to what extent specific clinical diagnostic groups were differentially impacted by different phases of the pandemic [12, 13]. Monitoring trends in psychiatric diagnoses is important not only because post-COVID psychiatric symptoms commonly persist for a long time [14, 15], but they may worsen the quality of life and increase mortality among COVID-19 infected patients [3, 16].

The availability of data from electronic health records (EHRs) on patients with pre-COVID psychiatric diagnoses provides an opportunity to improve our understanding of trends in post-COVID clinical psychiatric diagnoses. EHRs capture pre-COVID and post-COVID psychiatric diagnoses in patients, and their different levels of severity of COVID-19 infection (as defined by hospitalizations after testing positive), allowing comparisons of post-COVID psychiatric diagnoses in patients with and without COVID-19 infection/hospitalizations during the same period.

New York City (NYC) offers an important context for studying trends of post-COVID clinical psychiatric diagnoses. NYC was an epicenter of COVID-19 infections in the United States during the early phase of the pandemic [17]. In April 2020, results from NYC Health Opinion Poll (NYC HOP) [18] suggested that 52% of NYC residents reported COVID-19 related anxiety symptoms, which was 5 to 7 times higher than the pre-COVID anxiety levels among NYC residents (9% based on the 2018 NYC Community Health Survey [19] and 7% based on the 2017 NYC Social Determinants of Health Survey [20]). A year after the pandemic (August 2021), the same survey reported anxiety (25%) and depressive (18%) symptoms remained high [21]. In June 2022, results from the Community Health Needs Assessment (CHNA) still reported difficulty accessing mental health treatment among communities served by NYC Health + Hospitals [22]. Despite the warning signs of increasing psychiatric illness burden since the pandemic [7, 23], few studies have comprehensively assessed the trends of post-COVID clinical psychiatric diagnoses across the NYC metropolitan region [24].

**Fig. 1:**
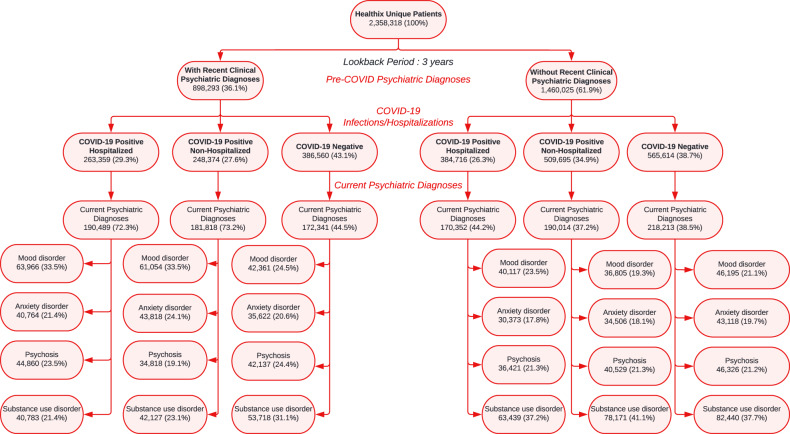
Flow chart of data extraction and study sample.

This study aimed to examine temporal trends in post-COVID clinical psychiatric diagnoses (after patients’ first SARS-CoV-2 test) between March 2020 and August 2021 (see Fig. 1). We are one of the first to differentiate trends by (1) pre-COVID psychiatric diagnoses (i.e., up to 3 years before the first SARS-CoV-2 test), (2) COVID-19 infection and hospitalization, and (3) psychiatric diagnosis groups. Before performing the analyses, we hypothesized that there would be greater post-COVID clinical psychiatric diagnoses among (1) patients with pre-COVID recent clinical psychiatric diagnoses, compared with those without; and (2) COVID-19 positive and/or hospitalized patients, compared with COVID-19 negative patients. Based on the earlier studies [25–27], we further hypothesized that (3) post-COVID trends in clinical psychiatric diagnoses would vary among people with different types of psychiatric diagnosis groups.

## Methods

### Study design and population

This is a time-series study with annual quarters (three months per quarter) as the primary unit of analysis. We used EHRs from Healthix, the largest health information exchange in the US, connecting over 30,000 healthcare providers across the NYC metropolitan region. Our sample came from 8,638 healthcare facilities across 5 boroughs (Bronx, Brooklyn, Manhattan, Queens, and Staten Island) in NYC and 192 unique zip codes (see eFig. 1 for geographic distribution). The healthcare facilities include 1783 (20.64%) ambulatory surgical centers, 1259 (14.58%) acute care units, 1192 (13.80%) non-intensive care units, 974 (11.28%) imaging and radiology centers, 947 (10.96%) medical offices, 758 (8.78%) hospice homes, 683 (7.91%) urgent care, 674 (7.80%) nursing homes, and 368 (4.26%) intensive care units.

We identified eligible patients with a SARS-CoV-2 test result recorded in Healthix between March 1, 2020, and August 31, 2021. We used March 1, 2020, as the start date because it was the first date after the US Food and Drug Administration (FDA) expanded laboratory-developed COVID-19 tests [28, 29], including real-time reverse transcription-polymerase chain reaction (RT-PCR) assays of nasopharyngeal, oropharyngeal, and sputum samples. In our sample, the RT-PCR test accounted for 23% of total COVID-19 tests, followed by Nucleic Acid Amplification Test (22%), antigen test (21%), antibody (serology) test (18%), and pooled sample testing (16%). This study was approved by the institutional review board of Weill Cornell Medicine (IRB protocol# 20-04021992).

### Data collection and measures

Figure 1 presents our three-step data extraction process. First, we identified two groups of patients with and without any recent clinical psychiatric diagnoses up to 3 years before their first COVID-19 test and before March 2020. Second, we identified three subgroups by COVID-19 infection and severity: (1) COVID-19 positive hospitalized, (2) COVID-19 positive non-hospitalized, and (3) COVID-19 negative. We used a heuristic search algorithm to classify COVID-19 infections (positive, negative) among patients with multiple COVID-19 lab tests. Healthix provided a contextual patient classification system, where we applied a Knuth-Morris-Pratt (KMP) algorithm [30] to match the patterns of lab test results and to separate COVID-19 positive and negative patients. KMP algorithm can effectively filter data and extract the required features for classification [31]. Third, for all eligible patients with COVID-19 lab test results, we extracted their subsequent clinical psychiatric diagnoses using diagnosis codes after the encounter index date of their COVID-19 tests.

We focused on four groups of clinical psychiatric diagnoses (eTable 1), consistent with the Agency for Healthcare Research and Quality (AHRQ) Clinical Classifications Software (CCS) categories and the *International Statistical Classification of Diseases, Tenth Revision*, including (1) anxiety disorders, (2) mood disorders, (3) psychosis, and (4) substance use disorder (SUD) [32]. The AHRQ-CCS has been widely used for examining clinical psychiatric diagnoses [33]. We chose these four clinical psychiatric diagnoses as they were associated with COVID-19 infections and hospitalizations in the early pandemic [16, 34] and in previous studies [7, 8].

Our primary outcome was the percentages of patients with a clinical psychiatric diagnosis among the total number of visits. We assessed patient-level demographic characteristics: age, sex, race, and ethnicity. Race and ethnicity were categorized using AHRQ race categories (i.e., American Indian or Alaska Native, Asian, Black or African American, Hispanic, Native Hawaiian or Other Pacific Islander, Other Race, Patient Declined to Answer, Unknown, White). Healthix normalizes race and ethnicity values to maximize the accuracy of race and ethnicity. To maintain the accuracy of the race and ethnicity variables, we assigned missing and uncertain codes for race and ethnicity to an “Unknown” category (eTable 2).

### Statistical analysis

To derive percentages of clinical psychiatric diagnoses per month adjusted for the number of visits per subgroup of interest in a specific month, we first obtained the monthly number of patients by COVID-19 infection and hospitalization status, separately by patients with and without recent clinical psychiatric diagnoses. We counted the number of distinct patients with specific clinical psychiatric diagnoses each month, divided by the number of patients enrolled in the same month. Monthly percentages of patients with clinical psychiatric diagnoses were examined following their first COVID-19 test (eTable 3, heat maps showing from lower [blue] to higher [red] rates).

Descriptive statistics were used to report percentages of clinical psychiatric diagnoses by month. We performed generalized linear mixed-effects (GLM) models with the Gaussian family and identified link specifications to estimate trends in clinical psychiatric diagnosis and their associations with recent clinical psychiatric diagnoses and COVID-19 infection and hospitalization status. In the primary analyses, we divided three months into a quarter and treated them as fixed effects. We used a significance threshold of *p* < 0.05 (2-sided). We provided results using listwise deletion, consistent with the descriptive analyses. For sensitivity analysis, we repeated the GLM by treating time as continuous measures (by month, by quarter) and applied multiple imputations to handle missing data. Results were consistent with the primary analyses reported below. Data extractions were conducted using the Python programming language version 3.7.4 (Python Software Foundation), and statistical analyses were completed using Stata 17/MP [StataCorp].

## Results

### Patient demographic characteristics

Of the total 2,358,318 eligible patients identified between March 1, 2020 and August 31, 2021, 271,345 (11.51%) were aged 0–17 years, 407,114 (17.26%) were aged 18–44 years, 827,280 (35.08%) patients were aged 46–65 years, 587,803 (24.92%) were aged 65–74 years, and 264,776 (11.23%) were 75 years and over. Most patients were females (*n* = 1,276,249, 54.12%). Among patients who reported a known race and ethnicity category, 103,295 (4.38%) were Asian Americans, 235,832 (10.00%) were Black, 296,308 (12.56%) were Hispanic, and 80,419 (3.41%) were White. 1,619,693 (68.68%) patients reported an “unknown” race category or “declined to answer” questions regarding race (Table 1).

**Table 1:** Demographic characteristics (*n* = 2,358,318).

Demographic characteristics	Total 2,358,318 (100%)	With recent clinical psychiatric diagnoses			Without recent clinical psychiatric diagnoses		
		898,293 (38.09%)			1,460,025 (61.91%)		
		COVID-19 positive hospitalized	COVID-19 positive non-hospitalized	COVID-19 negative	COVID-19 positive hospitalized	COVID-19 positive non-hospitalized	COVID-19 negative
		263,359 (29.32%)	248,374 (27.65%)	386,560 (43.03%)	384,716 (26.35%)	509,695 (34.91%)	565,614 (38.74%)
Age							
0–17 years old	271,345 (11.51%)	23,281 (8.84%)	23,297 (9.38%)	43,449 (11.24%)	41,665 (10.83%)	69,064 (13.55%)	70,589 (12.48%)
18–44 years old	407,114 (17.26%)	43,375 (16.47%)	53,053 (21.36%)	76,230 (19.72%)	67,018 (17.42%)	85,425 (16.76%)	82,014 (14.50%)
45–64 years old	827,280 (35.08%)	110,690 (42.03%)	99,474 (40.05%)	1,36,533 (35.32%)	1,30,842 (34.01%)	1,61,675 (31.72%)	1,88,067 (33.25%)
65–74 years old	587,803 (24.92%)	65,129 (24.73%)	56,455 (22.73%)	98,032 (25.36%)	1,05,489 (27.42%)	1,24,518 (24.43%)	1,38,180 (24.43%)
75+ years old	264,776 (11.23%)	20,884 (7.93%)	16,095 (6.48%)	32,316 (8.36%)	39,703 (10.32%)	69,013 (13.54%)	86,765 (15.34%)
Total observed	2,358,318 (100.00%)	2,63,359 (100.00%)	2,48,374 (100.00%)	3,86,560 (100.00%)	3,84,716 (100.00%)	5,09,695 (100.00%)	5,65,614 (100.00%)
Sex							
Male	1,082,069 (45.88%)	1,19,354 (45.32%)	1,18,077 (47.54%)	1,87,134 (48.41%)	1,62,927 (42.35%)	2,31,962 (45.51%)	2,62,615 (46.43%)
Female	1,276,249 (54.12%)	1,44,005 (54.68%)	1,30,297 (52.46%)	1,99,426 (51.59%)	2,21,789 (57.65%)	2,77,733 (54.49%)	3,02,999 (53.57%)
Total observed	2,358,318 (100.00%)	2,63,359 (100.00%)	2,48,374 (100.00%)	3,86,560 (100.00%)	3,84,716 (100.00%)	5,09,695 (100.00%)	5,65,614 (100.00%)
Race							
American Indian or Alaska Native	12,027 (0.51%)	2547 (0.97%)	2173 (0.87%)	1894 (0.49%)	2174 (0.57%)	1756 (0.34%)	1483 (0.26%)
Asian	103,295 (4.38%)	17,333 (6.58%)	16,011 (6.45%)	17,109 (4.43%)	17,849 (4.64%)	17,925 (3.52%)	17,067 (3.02%)
Black or African American	235,832 (10.00%)	37,333 (14.18%)	39,148 (15.76%)	42,509 (11.00%)	40,849 (10.62%)	37,925 (7.44%)	38,067 (6.73%)
Native Hawaiian or Other Pacific Islander	14,386 (0.61%)	2404 (0.91%)	2702 (1.09%)	2100 (0.54%)	2565 (0.67%)	2285 (0.45%)	2329 (0.41%)
Other Race	292,667 (12.41%)	49,962 (18.97%)	49,344 (19.87%)	48,892 (12.65%)	47,697 (12.40%)	47,789 (9.38%)	48,983 (8.66%)
Patient Declined to Answer	33,724 (1.43%)	5454 (2.07%)	5709 (2.30%)	5572 (1.44%)	5790 (1.51%)	5695 (1.12%)	5503 (0.97%)
Unknown	1,585,969 (67.25%)	1,32,984 (50.50%)	117,653 (47.37%)	2,55,967 (66.22%)	2,55,014 (66.29%)	38,5911 (75.71%)	4,38,440 (77.52%)
White	80,419 (3.41%)	15,342 (5.83%)	15,633 (6.29%)	12,517 (3.24%)	12,777 (3.32%)	10,409 (2.04%)	13,741 (2.43%)
Total observed	2,358,318 (100.00%)	2,63,359 (100.00%)	2,48,374 (100.00%)	3,86,560 (100.00%)	3,84,716 (100.00%)	5,09,695 (100.00%)	5,65,614 (100.00%)
Ethnicity							
Hispanic or Latino	296,308 (12.56%)	43,244 (16.42%)	28,389 (11.43%)	43,759 (11.32%)	47,512 (12.35%)	70,338 (13.80%)	63,066 (11.15%)
Non-Hispanic or Latino	303,571 (12.87%)	26,863 (10.20%)	23,148 (9.32%)	54,969 (14.22%)	50,859 (13.22%)	61,928 (12.15%)	85,804 (15.17%)
Patient Declined to Answer	442,115 (18.75%)	58,597 (22.25%)	58,045 (23.37%)	85,198 (22.04%)	79,213 (20.59%)	79,614 (15.62%)	81,448 (14.40%)
Unknown	1,316,324 (55.82%)	1,34,655 (51.13%)	1,38,792 (55.88%)	2,02,635 (52.42%)	2,07,131 (53.84%)	2,97,815 (58.43%)	3,35,296 (59.28%)
Total observed	2,358,318 (100.00%)	2,63,359 (100.00%)	2,48,374 (100.00%)	3,86,561 (100.00%)	3,84,715 (100.00%)	5,09,695 (100.00%)	5,65,614 (100.00%)

Recent clinical psychiatric diagnoses are defined as psychiatric diagnoses up to 3 years before the patient’s first COVID-19 reverse transcription-polymerase chain reaction (RT-PCR) test after March 2020.

Patients aged 45–64 years had higher percentages of hospitalization due to COVID-19 if they had a recent clinical psychiatric diagnosis (110,690 [42.03%]) compared to those aged 45–64 years but without a recent clinical psychiatric diagnosis (130,842 [34.01%]). Among patients with recent clinical psychiatric diagnoses, male and Hispanic patients had higher percentages of testing positive for COVID-19 and being hospitalized than females and non-Hispanic patients, respectively (Table 1).

### COVID-19 infection and hospitalization status and subsequent clinical psychiatric diagnoses by recent clinical psychiatric diagnoses

Of patients with a recent psychiatric diagnosis (Fig. 1), 511,733 (56.9%) were tested positive for COVID-19, (263,359 [29.32%] were hospitalized, 248,374 [27.65%] were non-hospitalized) and 386,560 (43.1%) patients tested negative. COVID-19 positive patients had more subsequent psychiatric diagnoses than patients with negative test results. Mood disorders were most commonly diagnosed among COVID-19 positive patients, either hospitalized (63,966 [33.6%]) or not hospitalized (61,054 [33.6%]), while SUD was the most common disorder among patients who tested negative (53,718 [31.2%]). eTable 4 shows the pre-COVID percentages (before March 2010). Post-pandemic percentages of clinical psychiatric diagnoses in the first six months were greater than pre-COVID percentages between January and March 2020 and between 2017 and 2019 among patients with recent clinical psychiatric diagnoses.

In contrast, among patients without recent clinical psychiatric diagnoses, more COVID-19 positive patients were non-hospitalized (509,695 [34.91%]) than hospitalized (384,716 [26.3%]). Compared to patients with recent clinical psychiatric diagnoses, those without recent pre-COVID clinical psychiatric diagnoses had lower percentages of subsequent clinical psychiatric diagnoses, and the highest percentages of clinical psychiatric diagnoses were SUD (Fig. 1).

### Overall trends of COVID-19 infection and hospitalization status by recent clinical psychiatric diagnoses

**Fig. 2:**
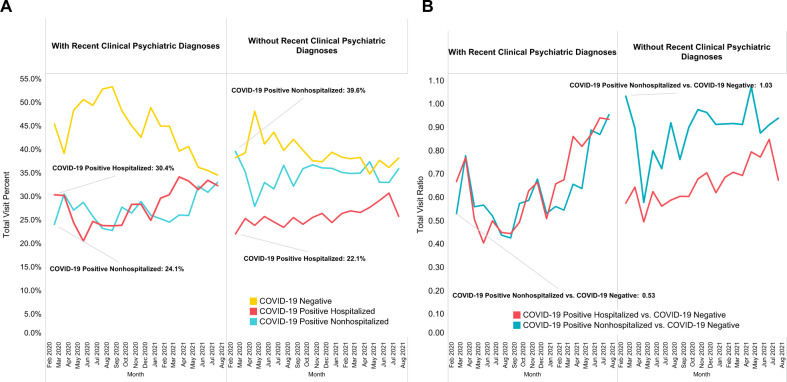
Trends of COVID-19 infection and hospitalization status by recent clinical psychiatric diagnoses. **A** Comparing total visit percentage. **B** Comparing total visit ratio.

Figure 2 compares trends of COVID-19 infection and hospitalizations by recent clinical psychiatric diagnoses up to three years before patients’ first SARS-Cov-2 PCR test. Among patients with pre-COVID clinical psychiatric diagnoses, in the first month of the pandemic (March 2020), more patients who tested positive were hospitalized (30.4%) than non-hospitalized (24.1%). In contrast, among patients without recent clinical psychiatric diagnoses, more COVID-19 positive patients were non-hospitalized (39.6%) than hospitalized (22.1%) in March 2020 (Fig. 2A). The number of patients with COVID-19 infection and hospitalizations increased compared to COVID-19 negative patients (Fig. 2B).

### Trends of clinical psychiatric diagnoses by recent clinical psychiatric diagnoses, separated by COVID-19 infection and hospitalization status

**Fig. 3:**
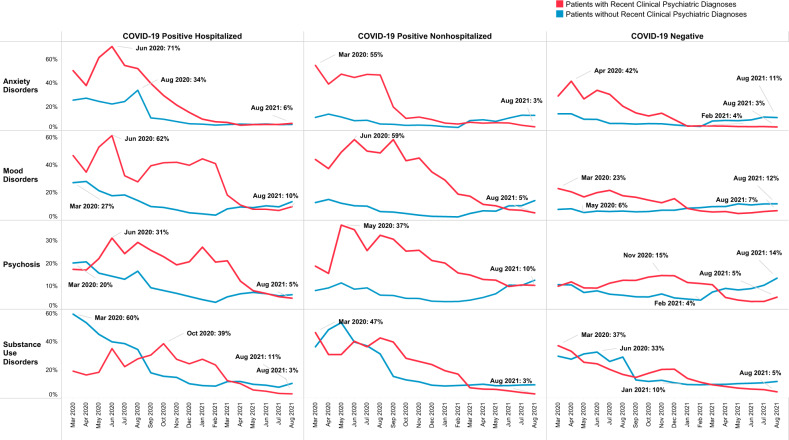
Trends of clinical psychiatric diagnoses by recent clinical psychiatric diagnoses, separated by COVID-19 infection and hospitalization status.

As shown in Fig. 3, patients with a recent clinical psychiatric diagnosis had greater anxiety disorders, mood disorders, and psychosis diagnoses in the early phase of the pandemic (March–August 2020) than those without recent clinical psychiatric diagnoses. These trends remained elevated for nearly a year till March 2021 among both COVID-19 positive and negative patients, except there were greater percentages of psychosis among patients without recent clinical psychiatric diagnoses in the first month of the pandemic (March–April 2020) among COVID-19 positive hospitalized patients.

In contrast, SUD diagnoses had the highest percentages among patients without recent clinical psychiatric diagnoses during the first six months of the pandemic (March-August 2020) among COVID-19 positive hospitalized patients (April 2020) and COVID-19 positive non-hospitalized patients and COVID-19 negative patients (June 2020).

SUD diagnoses decreased substantially between August-September 2020 among patients without a recent clinical psychiatric diagnosis but increased among those with a recent clinical psychiatric diagnosis. Yet, patients without a recent clinical psychiatric diagnosis had increased percentages of new psychiatric diagnoses starting February 2021 among both COVID-19 positive and negative patients. The largest magnitude of increases in post-COVID psychiatric diagnoses occurred in COVID-19 positive non-hospitalized patients (eTable 4).

Table 2 reports the associations between recent clinical psychiatric diagnoses, COVID-19 infection and hospitalization status, and trends in post-COVID psychiatric diagnosis. Compared to patients without a recent clinical psychiatric diagnosis, those with a recent clinical psychiatric diagnosis had increasing trends in subsequent diagnoses of anxiety disorders (*β* = 11.8, 95% CI [8.62, 14.9], *p* < 0.001), mood disorders (*β* = 16.8, 95% CI [13.4, 20.2], *p* < 0.001), and psychosis (*β* = 8.94, 95% CI [7.21, 10.7], *p* < 0.001). Compared to patients with a negative COVID-19 test, COVID-19 patients had greater subsequent diagnoses of all psychiatric diagnostic groups over time (*p* < 0.001).

**Table 2:** Associations between post-COVID clinical psychiatric diagnoses, pre-COVID clinical psychiatric diagnoses, and COVID-19 infection.

Characteristics	Anxiety disorders			Mood disorders			Psychosis			Substance use disorders		
	*β*	95% CI	*P*	*β*	95% CI	*P*	*β*	95% CI	*P*	*β*	95% CI	*P*
Time												
Mar–May 2020	1 [Reference]			1 [Reference]			1 [Reference]			1 [Reference]		
Jun–Aug 2020	−0.74	[−6.03, 4.54]	0.783	−1.98	[−7.71, 3.74]	0.497	1.41	[−1.50, 4.32]	0.342	−3.58	[−7.88, 0.72]	0.102
Sep–Nov 2020	−16.8***	[−22.1, −11.5]	<0.001	−4.88+	[−10.6, 0.85]	0.095	−1.48	[−4.39, 1.42]	0.317	−15.0***	[−19.2, −10.7]	<0.001
Dec 2020–Feb 2021	−23.7***	[−29.0, −18.4]	<0.001	−9.84***	[−15.6, −4.11]	0.001	−4.19**	[−7.10, −1.29]	0.005	−20.6***	[−24.9, −16.3]	<0.001
Mar–May 2021	−23.3***	[−28.6, −18.0]	<0.001	−16.3***	[−22.0, −10.5]	<0.001	−6.28***	[−9.19, −3.37]	<0.001	−26.2***	[−30.5, −21.9]	<0.001
Jun–Aug 2021	−22.7***	[−28.6, −16.8]	<0.001	−16.8***	[−23.2, −10.4]	<0.001	−7.48***	[−10.7, −4.24]	<0.001	−28.1***	[−32.9, −23.3]	<0.001
Recent clinical psychiatric diagnoses												
Without recent clinical psychiatric diagnoses	1 [Reference]			1 [Reference]			1 [Reference]			1 [Reference]		
With recent clinical psychiatric diagnoses	11.8***	[8.62, 14.9]	<0.001	16.8***	[13.4, 20.2]	<0.001	8.94***	[7.21, 10.7]	<0.001	−0.23	[−2.78, 2.33]	0.861
COVID-19 infection and hospitalization												
COVID positive negative	1 [Reference]			1 [Reference]			1 [Reference]			1 [Reference]		
COVID positive nonhospitalized	3.40+	[−0.45, 7.25]	0.083	9.89***	[5.72, 14.1]	<0.001	5.52***	[3.41, 7.64]	<0.001	5.26***	[2.13, 8.39]	0.001
COVID positive hospitalized	9.16***	[5.31, 13.0]	<0.001	11.8***	[7.60, 15.9]	<0.001	6.01***	[3.90, 8.13]	<0.001	4.54**	[1.41, 7.66]	0.004

Recent clinical psychiatric diagnoses are defined as psychiatric diagnoses up to 3 years before the patient’s first COVID-19 reverse transcription-polymerase chain reaction (RT-PCR) test after March 2020. Patients with clinical psychiatric diagnoses were aggregated by quarterly based observations.

### Trends of clinical psychiatric diagnoses by COVID-19 infection and hospitalization status, separated by recent clinical psychiatric diagnoses

**Fig. 4:**
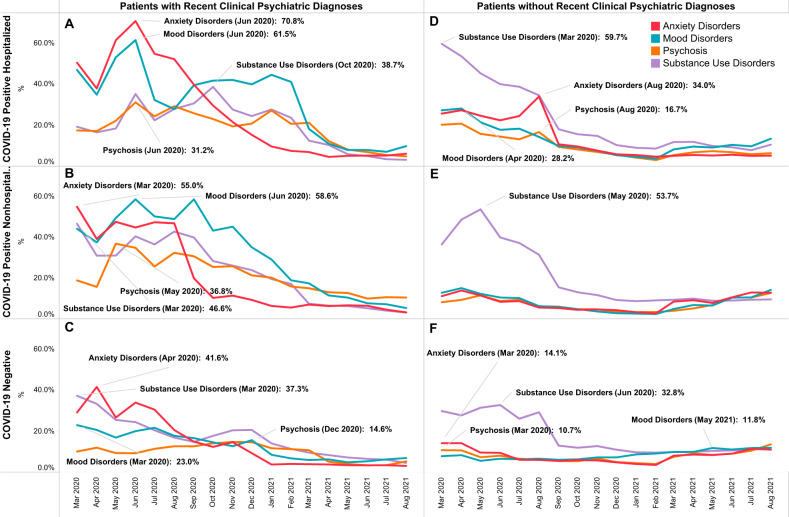
Trends of clinical psychiatric diagnoses by COVID-19 infection and hospitalization status, separated by recent clinical psychiatric diagnoses. **A** Trends in patients with recent psychiatric diagnoses and COVID-19 positive with hospitalization. **B** Trends in patients with recent psychiatric diagnoses and COVID-19 positive nonhospitalized. **C** Trends in patients with recent psychiatric diagnoses and COVID-19 negative. **D** Trends in patients without recent psychiatric diagnoses and COVID-19 positive with hospitalization. **E** Trends in patients without recent psychiatric diagnoses and COVID-19 positive nonhospitalized. **F** Trends in patients without recent psychiatric diagnoses and COVID-19 negative.

As shown in Fig. 4, among patients with recent clinical psychiatric diagnoses, during the first month of the pandemic (March 2020 to April 2020), all psychiatric diagnoses decreased among COVID-19 positive hospitalized and non-hospitalized patients (Fig. 4A, B), while anxiety disorders increased among COVID-19 negative patients (Fig. 4C).

Among COVID-19 positive hospitalized patients (Fig. 4A), percentages of anxiety disorder diagnoses were the highest until September 2020. The peak of anxiety disorders (70.8%) and mood disorders (61.5%) occurred in June 2020. Anxiety disorder diagnoses decreased afterward, whereas mood disorders re-emerged in September 2020 and remained the most prevalent until March 2021. Among COVID-19 positive non-hospitalized patients (Fig. 4B), anxiety disorder diagnoses were the highest between March and April 2020, while mood disorder diagnoses were the highest between May 2020 to March 2021.

Among COVID-19 negative patients (Fig. 4C), percentages of clinical psychiatric diagnoses each month were lower than those in COVID-19 positive patients. Clinical psychiatric diagnoses were the highest in the first two months of the pandemic for anxiety disorders (April 2020, 41.6%), mood disorders (March 2020, 23.0%), and SUD (January March, 37.3%), which decreased gradually. Psychosis diagnosis increased since October 2020 and remained the highest between October 2020 and February 2021 (peak in December 2020, 14.6%) before a gradual decrease till August 2021.

Among patients without a recent clinical psychiatric diagnosis (Fig. 4D, E), newly diagnosed SUD was substantially higher than diagnoses of anxiety disorders, mood disorders, and psychosis between March 2020 and August 2020, regardless of COVID-19 infection/hospitalization. Such high-level of SUD diagnoses started to decrease from August 2020 but remained the highest psychiatric diagnostic group till January 2021 (for COVID-19 negative patients), March 2020 (for non-hospitalized COVID-19 patients), and May 2021 (for hospitalized COVID-19 positive patients). By contrast, anxiety disorders, mood disorders, and psychosis began to increase after a year of the pandemic (since March 2021) among COVID-19 negative patients and COVID-19 positive non-hospitalized patients.

## Discussion

Patients with recent pre-COVID clinical psychiatric diagnoses, compared to those without, had greater percentages of anxiety disorders, mood disorders, and psychosis diagnoses, early in the pandemic in March 2020 in NYC. Anxiety and mood disorders were the most commonly diagnosed in the first year of the pandemic among COVID-19 positive patients with recent clinical psychiatric diagnoses. Among patients without a recent pre-COVID clinical psychiatric diagnosis, new SUD diagnoses were substantially higher than other psychiatric diagnoses in the first six months of the pandemic (March-August 2020), which remained the most diagnosed disorder till May 2021. Since February 2021, there have been continuous increases in newly diagnosed anxiety disorders, mood disorders, and psychosis across COVID-19 positive and negative patients.

### Differential trends by recent pre-COVID clinical psychiatric diagnoses

Our findings are consistent with previous studies suggesting increases in clinical psychiatric diagnoses after the COVID-19 pandemic [35–37]. We extend previous findings with systematic documentation of recent clinical psychiatric diagnoses up to 3 years before the pandemic, allowing benchmark comparisons of trends in post-COVID psychiatric diagnoses by preexisting psychiatric conditions [38, 39]. We further observed that among patients without recent clinical psychiatric diagnoses, SUD diagnoses were substantially high in the early pandemic between March and August 2020.

Individuals with pre-COVID clinical psychiatric diagnoses may be at greater risk for COVID-19 infection and hospitalization, partly because they were more likely to experience co-occurring medical conditions (e.g., cancer, cerebrovascular diseases, congestive heart failure, pulmonary disease) [40]. After being infected by SARS-CoV-2, patients with pre-COVID clinical psychiatric diagnoses, compared with those without, may have greater risks for subsequent psychiatric disorder diagnoses because of greater levels of stress, dysregulated immune functions [41], and social and economic consequences [3, 35].

In early March 2020, NYC emerged as an epicenter for COVID-19 [42], which exerted sudden and widespread closures of medical practices, disrupting access to mental health services for patients with pre-existing clinical psychiatric diagnoses [43]. The unprecedented social distancing policies also introduced stress, fear, and uncertainty that may have disproportionately impacted patients with pre-COVID clinical psychiatric diagnoses [35, 37, 44].

### Differential trends by COVID-19 infections and severity

Previous studies suggested that COVID-19 positive patients who survived are at increased risk of psychiatric sequelae [1]. We extended this line of research by demonstrating short-term and long-term (18 months) changes in psychiatric diagnoses across different stages of the pandemic [45]. For all patients, regardless of COVID-19 infection, anxiety and mood disorders diagnoses remained high in the first six months of the pandemic, which may have reflected the secondary impacts of the COVID-19 pandemic on mental health (e.g., through lockdown, social distancing, economic disruptions, grief, and loss) [11, 46]. The monthly percentages of psychiatric diagnoses decreased during the first month (March-April 2020) in COVID-19 positive patients, which may be explained by larger representations of 45–64 years old, female, Black, and Hispanic patients who tested positive for COVID-19, but who experienced barriers to accessing healthcare facilities for psychiatric treatment and diagnoses during the peak of COVID-19 infection in NYC [47, 48].

Similar to a cross-sectional study in Italy [49], we observed increases in psychiatric diagnoses were the highest early in the pandemic (when COVID-19 infections were the highest) but started to decrease along with the decline in COVID-19 cases. However, we further observed that among patients without pre-COVID psychiatric diagnoses, new psychiatric diagnoses increased after a year of the pandemic among all patients, which is consistent with a prior longitudinal study [8]. This may reflect the lagged impact of pandemic-related stress and trauma, losses of loved ones, disrupted healthcare utilizations, and social restrictions, which may emerge during the pandemic recovery phase [11, 50, 51]. Future studies are encouraged to investigate the compound impact of COVID-19 infections, pandemic-related stressors, and grief on clinical psychiatric diagnoses in the longer term [11].

### Differential trends by specific clinical psychiatric diagnostic groups

The dramatic increases in anxiety disorders and mood disorders diagnoses in the early pandemic are consistent with findings in the general population from national surveys in the US and other countries during the COVID-19 pandemic [52–55]. For example, the Johns Hopkins COVID-19 Civic Life and Public Health Survey covering 97% of national adults found greater symptoms of psychological distress in April 2020 than in 2018 [56]. It is noted that EHR data underestimate the number of psychiatric disorders because patients may face challenges in accessing timely mental health services or delayed seeking care for psychiatric illnesses during the pandemic to avoid COVID-19 infection risks in medical settings.

The dramatic increase in new SUD diagnoses among patients without recent clinical psychiatric diagnoses is consistent with prior investigations [8]. A nationwide survey conducted between April and May 2020 found an 18.2% increase in SUD among U.S. adults [57]. COVID-19 disruptions may also create barriers to access to therapy and compliance with medication for SUD patients [58]. Patients without recent psychiatric diagnosis may be at risk of substance misuse to cope with stress during the COVID-19 pandemic [57], which may be explained by insufficient access to harm reduction resources targeting people without pre-existing disorders.

The COVID-19 pandemic has posed challenges, as well as new opportunities for increasing access to treatment of opioid use disorder [59–61]. Recent studies found prescriptions for buprenorphine for SUD and opioid analgesics decreased among new, but not existing, patients during the COVID-19 pandemic [62]. In another time-series study conducted in Michigan, fatal overdose deaths showed a substantial increase in the spring of 2020 [63]. It is worth noting that medications for opioid use disorder (MOUD) and telehealth utilization were expanded throughout NYC during the pandemic, which may explain the continual decrease of post-COVID SUD diagnoses since August 2020 [64, 65]. As a majority of SUD may not be captured in clinical encounters, future studies are needed to empirically examine the compound mental health impact of the pandemic due to COVID-19 virus infections, treatment access, and government responses.

### Social determinants of psychiatric diagnoses

Other factors associated with the impact of the COVID-19 pandemic on clinical psychiatric diagnoses include social distancing, unemployment, school disruptions, economic downturn, and reduced mental health utilization [66]. Social determinants of health, including food insecurity [67], reduced exercises [68], comorbid medical conditions [69], sleep problems [70], and worsened interpersonal relationships [69] may also exacerbate the risks of psychiatric illnesses. Several studies have shown that COVID-19-associated outcomes have disproportionately impacted racial and ethnic minorities [71–74]. Among those with psychiatric diagnoses in our study, patients aged 45-64 years, females, those from unknown race/ethnicity groups, and Black and Asian patients were more likely to be infected by COVID-19 and hospitalized. Hence, future studies should focus on collecting more information on SDoH (e.g., economic disruptions, food insecurity, healthcare accessibilities) to understand the impacts of pandemic-related socioeconomic disruptions on clinical psychiatric diagnoses among vulnerable populations [71, 75, 76].

### Clinical and policy implications

Findings from this study could inform future pandemic-related policymaking. First, patients with recent clinical psychiatric diagnoses had higher anxiety and mood disorders than those without at the beginning of the pandemic. Because patients with recent clinical psychiatric diagnoses were associated with greater risks of COVID-19 infections and adverse outcomes [61], including suicide and mortality [40, 57, 75, 77–79], clinicians and public health practitioners need to prioritize screening and treating individuals with clinical psychiatric diagnoses as part of the strategy to control the pandemic while reducing disparities in access to healthcare support.

Second, given the substantial increase in SUD among patients without recent clinical psychiatric diagnoses, public health practitioners and policymakers should investigate rising substance misuse, including alcohol and opioids, and improve access to alternative modes of mental health care (i.e., telehealth or virtual care) [64, 80].

Third, the increase in new psychiatric diagnoses since February 2021 is of concern. Policymakers should continue investigating the adverse long-term mental health impacts of the COVID-19 pandemic and monitoring the effects of mental health treatment disruptions and reconnections during the later recovery from COVID-19. Comprehensive strategies to reduce psychiatric diagnoses should address pre-existing and pandemic-related SDoH.

### Strengths and limitations

One of the key strengths of our study is screening for pre-COVID psychiatric diagnoses (3 years before the first COVID-19 tests) and using a heuristic search algorithm to classify COVID-19 positive/negative among patients with multiple COVID tests. Understanding long-term trends in psychiatric sequelae over time across COVID-19 infection and severity provides a detailed understanding of the potential effects of COVID-19 infection on mental health, which could inform future interventions to reduce psychiatric diagnoses among specific at-risk populations.

This study has limitations. First, our sample was only from NYC, which may not be generalized to other areas in the US. Data from the Bronx and Staten Island is not comprehensive as those from the other boroughs. Clinical psychiatric diagnoses based on ICD-10 diagnosis codes may not reflect population rates of psychiatric illnesses and may not include lifetime psychiatric history, compared with clinical interviews in previous studies [7, 8]. Nevertheless, psychiatric diagnoses in EHRs reflect the clinically recognized prevalence of mental health disorders.

Second, there are substantial missing values in race and ethnicity data, which is a common challenge when using EHR data. We also lack individual-level socioeconomic status data, as in most of the other EHR datasets. Since existing socioeconomic and racial disparities in healthcare access in NYC may further exacerbate the adverse mental health impact of COVID-19 infections on clinical psychiatric diagnoses [47], future research should explore the associations between socioeconomic status and trends in clinical psychiatric diagnoses.

Third, although we show possible relationships between COVID-19 infections, hospitalizations, and clinical psychiatric diagnoses by recent clinical psychiatric diagnoses, we did not estimate their causal associations, which would require further adjustment of potential confounding variables.

Fourth, we do not know the mortality status of patients in Healthix or differentiate co-morbid clinical psychiatric diagnoses. We also included the relatively broad category of clinical psychiatric diagnoses instead of specifying subgroups within each diagnostic group. Future research is encouraged to study how subgroups of clinical psychiatric diagnoses may respond differently to COVID-19 infections, as they may have distinct biological underpinnings [81].

## Conclusion

Patients with recent clinical psychiatric diagnoses were at higher risk of mood disorders and anxiety at the beginning of the pandemic. Among patients without a recent clinical psychiatric diagnosis, SUD increased from March 2020 to September 2020, and subsequent anxiety, mood, and psychosis diagnoses increased after a year of the pandemic. Future policies should consider improving access to mental health treatment, expanding alternative services such as telehealth coverage, and developing targeted evidence-based mental health and substance use recovery interventions [64]. Policies that address pre-COVID social determinants of health, including food, health care, and socioeconomically disadvantaged communities, are also critical to reducing the social and mental health impact of COVID-19.

## Supplementary information

Supplementary Materials

## References

[1] Taquet M, Luciano S, Geddes JR, Harrison PJ (2021). Bidirectional associations between COVID-19 and psychiatric disorder: retrospective cohort studies of 62 354 COVID-19 cases in the USA. Lancet Psychiatry.

[2] Abel KM, Carr MJ, Ashcroft DM, Chalder T, Chew-Graham CA, Hope H (2021). Association of SARS-CoV-2 infection with psychological distress, psychotropic prescribing, fatigue, and sleep problems among UK primary care patients. JAMA Netw Open.

[3] Nishimi K, Neylan TC, Bertenthal D, Seal KH, O’Donovan A (2022). Association of psychiatric disorders with incidence of SARS-CoV-2 breakthrough infection among vaccinated adults. JAMA Netw Open.

[4] Venkatesan P (2021). NICE guideline on long COVID. Lancet Respir Med.

[5] Levine RL (2022). Addressing the long-term effects of COVID-19. JAMA.

[6] CDC. Post-COVID Conditions. Cent. Dis. Control Prev. 2022. https://www.cdc.gov/coronavirus/2019-ncov/long-term-effects/index.html (accessed 17 Sep 2022).

[7] Wang S, Quan L, Chavarro JE, Slopen N, Kubzansky LD, Koenen KC et al. Associations of Depression, Anxiety, Worry, Perceived Stress, and Loneliness Prior to Infection With Risk of Post–COVID-19 Conditions. JAMA Psychiatry. 2022. 10.1001/jamapsychiatry.2022.2640. 10.1001/jamapsychiatry.2022.2640 PMC9453634 36069885

[8] Murphy E, Svob C, Van Dijk M, Gameroff MJ, Skipper J, Abraham E, et al. The effects of the pandemic on mental health in persons with and without a psychiatric history. Psychol Med. 2021:1–9. 10.1017/S0033291721004372. 10.1017/S0033291721004372 PMC8632413 34743762

[9] McGrail DJ, Dai J, McAndrews KM, Kalluri R (2020). Enacting national social distancing policies corresponds with dramatic reduction in COVID19 infection rates. PLOS ONE.

[10] Chudik et al. Economic consequences of Covid-19: A counterfactual multi-country analysis. Voxeu. 2020 https://voxeu.org/article/economic-consequences-covid-19-multi-country-analysis.

[11] Xiao Y, Yip PS-F, Pathak J, Mann JJ Association of social determinants of health and vaccinations with child mental health during the COVID-19 pandemic in the US. JAMA Psychiatry. 2022 10.1001/jamapsychiatry.2022.0818. 10.1001/jamapsychiatry.2022.0818 PMC9047762 35475851

[12] Favreau M, Hillert A, Osen B, Gärtner T, Hunatschek S, Riese M (2021). Psychological consequences and differential impact of the COVID-19 pandemic in patients with mental disorders. Psychiatry Res.

[13] Demartini B, Nisticò V, D’Agostino A, Priori A, Gambini O. Early psychiatric impact of COVID-19 pandemic on the general population and healthcare workers in italy: a preliminary study. Front Psychiatry. 2020; **11**. 10.3389/fpsyt.2020.561345 (accessed 13 Sep2022). 10.3389/fpsyt.2020.561345 PMC7783153 33414728

[14] Xie Q, Liu X-B, Xu Y-M, Zhong B-L (2021). Understanding the psychiatric symptoms of COVID-19: a meta-analysis of studies assessing psychiatric symptoms in Chinese patients with and survivors of COVID-19 and SARS by using the Symptom Checklist-90-Revised. Transl Psychiatry.

[15] Liu X, Zhu M, Zhang R, Zhang J, Zhang C, Liu P (2021). Public mental health problems during COVID-19 pandemic: a large-scale meta-analysis of the evidence. Transl Psychiatry.

[16] Nemani K, Li C, Olfson M, Blessing EM, Razavian N, Chen J (2021). Association of psychiatric disorders with mortality among patients with COVID-19. JAMA Psychiatry.

[17] McKinley J New York City Region Is Now an Epicenter of the Coronavirus Pandemic. N. Y. Times. 2020. https://www.nytimes.com/2020/03/22/nyregion/Coronavirus-new-York-epicenter.html.

[18] Dobosh K, Tiberio J, Dongchung T. Inequities in New Yorkers’ Experiences of the COVID-19 Pandemic. New York City Department of Health and Mental Hygiene, 2021 https://www1.nyc.gov/assets/doh/downloads/pdf/epi/databrief130.pdf.

[19] Gwynn RC, McQuistion HL, McVeigh KH, Garg RK, Frieden TR, Thorpe LE (2008). Prevalence, diagnosis, and treatment of depression and generalized anxiety disorder in a diverse urban community. Psychiatr Serv.

[20] Tuskeviciute R, Hoenig J, Norman C. The social determinants of mental health among New York City adults. New York City Department of Health and Mental Hygiene, 2019 https://www1.nyc.gov/assets/doh/downloads/pdf/epi/databrief130.pdf.

[21] Magas I, Norman C. Impacts of COVID-19 on Mental Health in New York City, 2021. New York City Department of Health and Mental Hygiene, 2021 https://www1.nyc.gov/assets/doh/downloads/pdf/epi/databrief130.pdf.

[22] NYC Health + Hospitals. Community Health Needs Assessment 2022. 2022 https://hhinternet.blob.core.windows.net/uploads/2022/07/community-health-needs-asssessment-2022.pdf.

[23] Chen F, Yan W, Calhoun VD, Yu L, Chen L, Hao X (2022). A fast online questionnaire for screening mental illness symptoms during the COVID-19 pandemic. Transl Psychiatry.

[24] Anmella G, Arbelo N, Fico G, Murru A, Llach CD, Madero S (2020). COVID-19 inpatients with psychiatric disorders: Real-world clinical recommendations from an expert team in consultation-liaison psychiatry. J Affect Disord.

[25] Asmundson GJG, Paluszek MM, Landry CA, Rachor GS, McKay D, Taylor S (2020). Do pre-existing anxiety-related and mood disorders differentially impact COVID-19 stress responses and coping?. J Anxiety Disord.

[26] Solé B, Verdolini N, Amoretti S, Montejo L, Rosa AR, Hogg B (2021). Effects of the COVID-19 pandemic and lockdown in Spain: comparison between community controls and patients with a psychiatric disorder. Preliminary results from the BRIS-MHC STUDY. J Affect Disord.

[27] Hölzle P, Aly L, Frank W, Förstl H, Frank A (2020). COVID-19 distresses the depressed while schizophrenic patients are unimpressed: A study on psychiatric inpatients. Psychiatry Res.

[28] CDC. Testing Strategies for SARS-CoV-2. Cent. Dis. Control Prev. 2020. https://www.cdc.gov/coronavirus/2019-ncov/lab/resources/sars-cov2-testing-strategies.html (accessed 22 Sep2022).

[29] Sharfstein JM, Becker SJ, Mello MM (2020). Diagnostic testing for the novel coronavirus. JAMA.

[30] JavaPoint. DAA Knuth-Morris-Pratt Algorithm - javatpoint. www.javatpoint.com. 2022. https://www.javatpoint.com/daa-knuth-morris-pratt-algorithm (accessed 16 Sep2022).

[31] Gada V, Shegaonkar M, Inamdar M, Dinesh S, Sapariya D, Konde V (2022). Data analysis of COVID-19 hospital records using contextual patient classification system. Ann Data Sci.

[32] Agency for Healthcare Research and Quality. Clinical Classifications Software (CCS) for ICD-9-CM. 2017. https://www.hcup-us.ahrq.gov/toolssoftware/ccs/ccs.jsp (accessed 6 Apr2022).

[33] Ellis RP, Hsu HE, Song C, Kuo T-C, Martins B, Siracuse JJ (2020). Diagnostic category prevalence in 3 classification systems across the transition to the international classification of diseases, tenth revision, clinical modification. JAMA Netw Open.

[34] Hasin DS, Fink DS, Olfson M, Saxon AJ, Malte C, Keyes KM (2022). Substance use disorders and COVID-19: An analysis of nation-wide Veterans Health Administration electronic health records. Drug Alcohol Depend.

[35] Brooks SK, Webster RK, Smith LE, Woodland L, Wessely S, Greenberg N (2020). The psychological impact of quarantine and how to reduce it: rapid review of the evidence. Lancet.

[36] Wathelet M, Fovet T, Jousset A, Duhem S, Habran E, Horn M (2021). Prevalence of and factors associated with post-traumatic stress disorder among French university students 1 month after the COVID-19 lockdown. Transl Psychiatry.

[37] Jeong H, Yim HW, Song Y-J, Ki M, Min J-A, Cho J (2016). Mental health status of people isolated due to Middle East Respiratory Syndrome. Epidemiol Health.

[38] Pan K-Y, Kok AAL, Eikelenboom M, Horsfall M, Jörg F, Luteijn RA (2021). The mental health impact of the COVID-19 pandemic on people with and without depressive, anxiety, or obsessive-compulsive disorders: a longitudinal study of three Dutch case-control cohorts. Lancet Psychiatry.

[39] Ahrens KF, Neumann RJ, Kollmann B, Brokelmann J, von Werthern NM, Malyshau A (2021). Impact of COVID-19 lockdown on mental health in Germany: longitudinal observation of different mental health trajectories and protective factors. Transl Psychiatry.

[40] Li L, Li F, Fortunati F, Krystal JH (2020). Association of a prior psychiatric diagnosis with mortality among hospitalized patients with coronavirus disease 2019 (COVID-19) Infection. JAMA Netw Open.

[41] Banerjee D, Kosagisharaf JR, Sathyanarayana Rao TS (2021). ‘The dual pandemic’ of suicide and COVID-19: A biopsychosocial narrative of risks and prevention. Psychiatry Res.

[42] Uppal A, Silvestri DM, Siegler M, Natsui S, Boudourakis L, Salway RJ (2020). Critical Care And Emergency Department Response At The Epicenter Of The COVID-19 Pandemic: New York City’s public health system response to COVID-19 included increasing the number of intensive care units, transferring patients between hospitals, and supplementing critical care staff. Health Aff (Millwood).

[43] Tausch A, Souza RO e, Viciana CM, Cayetano C, Barbosa J, Hennis AJ. Strengthening mental health responses to COVID-19 in the Americas: A health policy analysis and recommendations. Lancet Reg Health – Am. 2022; 5 10.1016/j.lana.2021.100118. 10.1016/j.lana.2021.100118 PMC8782269 35098200

[44] Wellenius GA, Vispute S, Espinosa V, Fabrikant A, Tsai TC, Hennessy J (2021). Impacts of social distancing policies on mobility and COVID-19 case growth in the US. Nat Commun.

[45] Anderson KN, Radhakrishnan L, Lane RI, Sheppard M, DeVies J, Azondekon R et al. Changes and Inequities in Adult Mental Health–Related Emergency Department Visits During the COVID-19 Pandemic in the US. JAMA Psychiatry. 2022. 10.1001/jamapsychiatry.2022.0164. 10.1001/jamapsychiatry.2022.0164 PMC8928092 35293958

[46] Woolf SH, Chapman DA, Sabo RT, Weinberger DM, Hill L (2020). Excess deaths from COVID-19 and other causes, March-April 2020. Jama.

[47] Lieberman-Cribbin W, Tuminello S, Flores RM, Taioli E (2020). Disparities in COVID-19 testing and positivity in New York City. Am J Prev Med.

[48] Dalsania AK, Fastiggi MJ, Kahlam A, Shah R, Patel K, Shiau S (2022). The relationship between social determinants of health and racial disparities in COVID-19 mortality. J Racial Ethn Health Disparities.

[49] Lega I, Nisticò L, Palmieri L, Caroppo E, Noce CL, Donfrancesco C et al. Psychiatric disorders among hospitalized patients deceased with COVID-19 in Italy. eClinicalMedicine. 2021;35. 10.1016/j.eclinm.2021.100854. 10.1016/j.eclinm.2021.100854 PMC8062162 33907730

[50] Aknin LB, De Neve J-E, Dunn EW, Fancourt DE, Goldberg E, Helliwell JF et al. Mental health during the first year of the COVID-19 pandemic: a review and recommendations for moving forward. Perspect Psychol Sci. 2022: 17456916211029964. 10.1177/17456916211029964 PMC9274782 35044275

[51] Bernardini F, Attademo L, Rotter M, Compton MT (2021). Social determinants of mental health as mediators and moderators of the mental health impacts of the COVID-19 pandemic. Psychiatr Serv.

[52] Skoda E-M, Spura A, De Bock F, Schweda A, Dörrie N, Fink M (2021). Veränderung der psychischen Belastung in der COVID-19-Pandemie in Deutschland: Ängste, individuelles Verhalten und die Relevanz von Information sowie Vertrauen in Behörden. Bundesgesundheitsblatt - Gesundheitsforschung - Gesundheitsschutz.

[53] Xiong J, Lipsitz O, Nasri F, Lui LMW, Gill H, Phan L (2020). Impact of COVID-19 pandemic on mental health in the general population: A systematic review. J Affect Disord.

[54] Li LZ, Wang S (2020). Prevalence and predictors of general psychiatric disorders and loneliness during COVID-19 in the United Kingdom. Psychiatry Res.

[55] Ren X, Huang W, Pan H, Huang T, Wang X, Ma Y (2020). Mental health during the Covid-19 Outbreak in China: a Meta-Analysis. Psychiatr Q.

[56] McGinty EE, Presskreischer R, Han H, Barry CL (2020). Psychological distress and loneliness reported by US Adults in 2018 and April 2020. JAMA.

[57] McKnight-Eily LR, Okoro CA, Strine TW, Verlenden J, Hollis ND, Njai R (2021). Racial and ethnic disparities in the prevalence of stress and worry, mental health conditions, and increased substance use among adults during the COVID-19 Pandemic — United States, April and May 2020. MMWR Morb Mortal Wkly Rep..

[58] Haley DF, Saitz R (2020). The opioid epidemic during the COVID-19 pandemic. JAMA.

[59] Dowd WN, Mark TL (2022). Changes in buprenorphine prescribing to medicaid beneficiaries during the first year of the COVID-19 pandemic. JAMA Netw Open.

[60] Schmidt RA, Genois R, Jin J, Vigo D, Rehm J, Rush B (2021). The early impact of COVID-19 on the incidence, prevalence, and severity of alcohol use and other drugs: A systematic review. Drug Alcohol Depend.

[61] Wang QQ, Kaelber DC, Xu R, Volkow ND (2021). COVID-19 risk and outcomes in patients with substance use disorders: analyses from electronic health records in the United States. Mol Psychiatry.

[62] Currie JM, Schnell MK, Schwandt H, Zhang J (2021). Prescribing of opioid analgesics and buprenorphine for opioid use disorder during the COVID-19 pandemic. JAMA Netw Open.

[63] Cartus AR, Li Y, Macmadu A, Goedel WC, Allen B, Cerdá M (2022). Forecasted and Observed Drug Overdose Deaths in the US During the COVID-19 Pandemic in 2020. JAMA Netw Open.

[64] Jones CM, Shoff C, Hodges K, Blanco C, Losby JL, Ling SM et al. Receipt of telehealth services, receipt and retention of medications for opioid use disorder, and medically treated overdose among medicare beneficiaries before and during the COVID-19 pandemic. JAMA Psychiatry. 2022. 10.1001/jamapsychiatry.2022.2284. 10.1001/jamapsychiatry.2022.2284 PMC9434479 36044198

[65] Tofighi B, McNeely J, Walzer D, Fansiwala K, Demner A, Chaudhury CS (2022). A telemedicine buprenorphine clinic to serve New York City: Initial Evaluation of the NYC Public Hospital System’s Initiative to Expand Treatment Access During the COVID-19 Pandemic. J Addict Med.

[66] Benke C, Autenrieth LK, Asselmann E, Pané-Farré CA (2020). Lockdown, quarantine measures, and social distancing: Associations with depression, anxiety and distress at the beginning of the COVID-19 pandemic among adults from Germany. Psychiatry Res.

[67] Wolfson JA, Garcia T, Leung CW (2021). Food insecurity is associated with depression, anxiety, and stress: evidence from the early days of the COVID-19 pandemic in the United States. Health Equity.

[68] Hu S, Tucker L, Wu C, Yang L (2020). Beneficial effects of exercise on depression and anxiety during the Covid-19 Pandemic: a narrative review. Front Psychiatry.

[69] Hossain MM, Tasnim S, Sultana A, Faizah F, Mazumder H, Zou L (2020). Epidemiology of mental health problems in COVID-19: a review. F1000Research.

[70] Obuobi-Donkor G, Eboreime E, Shalaby R, Agyapong B, Oluwasina F, Adu M (2022). Evaluating the prevalence and predictors of moderate to severe depression in Fort McMurray, Canada during the COVID-19 Pandemic. Int J Environ Res Public Health.

[71] Acosta AM, Garg S, Pham H, Whitaker M, Anglin O, O’Halloran A (2021). Racial and Ethnic Disparities in Rates of COVID-19–Associated Hospitalization, Intensive Care Unit Admission, and In-Hospital Death in the United States From March 2020 to February 2021. JAMA Netw Open.

[72] Moore JT, Ricaldi JN, Rose CE, Fuld J, Parise M, Kang GJ (2020). Disparities in incidence of COVID-19 among underrepresented racial/ethnic groups in counties identified as hotspots during June 5–18, 2020—22 states, February–June 2020. Morb Mortal Wkly Rep..

[73] Lee FC, Adams L, Graves SJ, Massetti GM, Calanan RM, Penman-Aguilar A (2021). Counties with high COVID-19 incidence and relatively large racial and ethnic minority populations—United States, April 1–December 22, 2020. Morb Mortal Wkly Rep..

[74] Hillis, SD, Blenkinsop, A, Villaveces, A, Annor, FB, Liburd, L, Massetti, GM, et al. COVID-19–Associated Orphanhood and Caregiver Death in the United States. Pediatrics, 2021;148:e2021053760. 10.1542/peds.2021-053760. 10.1542/peds.2021-053760 PMC10896160 34620728

[75] Ogedegbe G, Ravenell J, Adhikari S, Butler M, Cook T, Francois F (2020). Assessment of racial/ethnic disparities in hospitalization and mortality in patients With COVID-19 in New York City. JAMA Netw Open.

[76] Tummalapalli SL, Silberzweig J, Cukor D, Lin JT, Barbar T, Liu Y (2021). Racial and neighborhood-level disparities in COVID-19 incidence among patients on hemodialysis in New York City. J Am Soc Nephrol.

[77] Pirkis J, Gunnell D, Shin S, Del Pozo-Banos M, Arya V, Aguilar PA (2022). Suicide numbers during the first 9-15 months of the COVID-19 pandemic compared with pre-existing trends: An interrupted time series analysis in 33 countries. eClinicalMedicine.

[78] Ko NY, Hong S, Winn RA, Calip GS (2020). Association of Insurance Status and Racial Disparities With the Detection of Early-Stage Breast Cancer. JAMA Oncol.

[79] Larson PS, Bergmans RS. Impact of the COVID-19 pandemic on temporal patterns of mental health and substance abuse related mortality in Michigan: An interrupted time series analysis. Lancet Reg Health – Am. 2022; 10. 10.1016/j.lana.2022.100218. 10.1016/j.lana.2022.100218 PMC8898171 35284903

[80] Myran DT, Cantor N, Rhodes E, Pugliese M, Hensel J, Taljaard M (2022). Physician health care visits for mental health and substance use during the COVID-19 Pandemic in Ontario, Canada. JAMA Netw Open.

[81] Pérez-Vigil A, Fernández de la Cruz L, Brander G, Isomura K, Jangmo A, Feldman I (2018). Association of obsessive-compulsive disorder with objective indicators of educational attainment: a nationwide register-based sibling control study. JAMA Psychiatry.

