# Supplementary material for: Trends in psychiatric diagnoses by COVID-19 infection and hospitalization among patients with and without recent clinical psychiatric diagnoses in New York city from March 2020 to August 2021
Source: Transl Psychiatry. 2022 Nov 21;12:492. doi: 10.1038/s41398-022-02255-8 (PMC9681844; doi:10.1038/s41398-022-02255-8)

# eTable 1 ICD-10 Codes Associated with Each Psychiatric Diagnostic Category and Comorbidities

| **AHRQ-CCS** | **Psychiatric Disorders** | **ICD-10 Code** | **Descriptions** |
| --- | --- | --- | --- |
| 651 | Anxiety disorders | F40* | Phobic anxiety disorders |
|  |  | F41* | Other anxiety disorders |
|  |  | F42* | Obsessive-compulsive disorder |
|  |  | F43* | Reaction to severe stress, and adjustment disorders |
|  |  | F44* | Dissociative and conversion disorders |
|  |  | F45* | Somatoform disorders |
|  |  | F48* | Other nonpsychotic mental disorders |
| 657 | Mood disorders | F30* | Manic episode |
|  |  | F31* | Bipolar disorder |
|  |  | F32* | Depressive episode |
|  |  | F33* | Major depressive disorder, recurrent |
|  |  | F34* | Persistent mood [affective] disorders |
|  |  | F39* | Unspecified mood [affective] disorder |
| 659 | Psychosis  (Schizophrenia and other psychotic disorders) | F20* | Schizophrenia |
|  |  | F21* | Schizotypal disorder |
|  |  | F22* | Delusional disorders |
|  |  | F23* | Brief psychotic disorder |
|  |  | F24* | Shared psychotic disorder |
|  |  | F25* | Schizoaffective disorders |
|  |  | F28* | Other psychotic disorder not due to a substance or known physiological condition |
|  |  | F29* | Unspecified psychosis not due to a substance or known physiological condition |
| 660-661 | Substance use disorders  (Alcohol-Related Disorders, Substance-Related Disorders) | F10* | Alcohol related disorders |
|  |  | F11* | Opioid related disorders |
|  |  | F12* | Cannabis related disorders |
|  |  | F13* | Sedative, hypnotic, or anxiolytic related disorders |
|  |  | F14* | Cocaine related disorders |
|  |  | F15* | Other stimulant related disorders |
|  |  | F16* | Hallucinogen related disorders |
|  |  | F17* | Nicotine dependence |
|  |  | F18* | Inhalant related disorders |
|  |  | F19* | Other psychoactive substance related disorders |

*Abbreviations*: AHRQ-CCS: Agency for Healthcare Research and Quality (AHRQ) Clinical Classifications Software (CCS)

# eTable 2 Missing data by demographic characteristics.

| **Demographic Characteristics** | **COVID-19 Status** | | | | | | **Pre-COVID Recent Clinical Psychiatric Diagnoses** | | | | **Psychiatric Disorders** | | | | | | | |
| --- | --- | --- | --- | --- | --- | --- | --- | --- | --- | --- | --- | --- | --- | --- | --- | --- | --- | --- |
|  | COVID-19 Hospitalization | | COVID-19 Positive Non-Hospitalization | | COVID-19 Negative | | Patient with Recent Clinical Psychiatric Diagnoses | | Patient without Recent Clinical Psychiatric Diagnoses | | Anxiety Disorders | | Mood Disorders | | Psychosis | | Substance Use Disorders | |
|  | Missing N | Missing % | Missing N | Missing % | Missing N | Missing % | Missing N | Missing % | Missing N | Missing % | Missing N | Missing % | Missing N | Missing % | Missing N | Missing % | Missing N | Missing % |
| **Age** |  |  |  |  |  |  |  |  |  |  |  |  |  |  |  |  |  |  |
| 0 - 17 years old | 7547 | 0.32% | 3066 | 0.13% | 5424 | 0.23% | 5896 | 0.25% | 8254 | 0.35% | 8018 | 0.34% | 5660 | 0.24% | 4245 | 0.18% | 5424 | 0.23% |
| 18 - 44 years old | 10612 | 0.45% | 2830 | 0.12% | 5424 | 0.23% | 3773 | 0.16% | 10141 | 0.43% | 5424 | 0.23% | 5424 | 0.23% | 10848 | 0.46% | 4952 | 0.21% |
| 45 - 64 years old | 15093 | 0.64% | 5424 | 0.23% | 6132 | 0.26% | 7547 | 0.32% | 5188 | 0.22% | 10141 | 0.43% | 4245 | 0.18% | 4245 | 0.18% | 3537 | 0.15% |
| 65 - 74 years old | 5424 | 0.23% | 3773 | 0.16% | 4481 | 0.19% | 5660 | 0.24% | 5424 | 0.23% | 12499 | 0.53% | 8726 | 0.37% | 5424 | 0.23% | 4481 | 0.19% |
| 75+ years old | 10141 | 0.43% | 3537 | 0.15% | 5188 | 0.22% | 3302 | 0.14% | 10612 | 0.45% | 10612 | 0.45% |  | 0.00% |  | 0.00% |  | 0.00% |
| **Sex** |  |  |  |  |  |  |  |  |  |  |  |  |  |  |  |  |  |  |
| Male | 54949 | 2.33% | 47402 | 2.01% | 49289 | 2.09% | 60373 | 2.56% | 83484 | 3.54% | 62967 | 2.67% | 67825 | 2.88% | 55185 | 2.34% | 49761 | 2.11% |
| Female | 55420 | 2.35% | 44572 | 1.89% | 49761 | 2.11% | 60514 | 2.57% | 102115 | 4.33% | 81362 | 3.45% | 93154 | 3.95% | 34903 | 1.48% | 75938 | 3.22% |
| **Race** |  |  |  |  |  |  |  |  |  |  |  |  |  |  |  |  |  |  |
| American Indian or Alaska Native | 30894 | 1.31% | 29243 | 1.24% | 24126 | 1.02% | 57779 | 2.45% | 83956 | 3.56% | 55420 | 2.35% | 56128 | 2.38% | 43393 | 1.84% | 49053 | 2.08% |
| Asian | 25470 | 1.08% | 29243 | 1.24% | 55185 | 2.34% | 58015 | 2.46% | 83956 | 3.56% | 81362 | 3.45% | 55420 | 2.35% | 55326 | 2.35% | 62260 | 2.64% |
| Black or African American | 45516 | 1.93% | 57779 | 2.45% | 34431 | 1.46% | 109426 | 4.64% | 83956 | 3.56% | 60373 | 2.56% | 60609 | 2.57% | 62495 | 2.65% | 51411 | 2.18% |
| Native Hawaiian or Other Pacific Islander | 54949 | 2.33% | 48110 | 2.04% | 57850 | 2.45% | 57779 | 2.45% | 105181 | 4.46% | 81362 | 3.45% | 70042 | 2.97% | 43393 | 1.84% | 66976 | 2.84% |
| Other Race | 48110 | 2.04% | 60373 | 2.56% | 55586 | 2.36% | 55369 | 2.35% | 81598 | 3.46% | 80183 | 3.40% | 81834 | 3.47% | 24762 | 1.05% | 41035 | 1.74% |
| Patient Declined to Answer | 69335 | 2.94% | 58015 | 2.46% | 45987 | 1.95% | 83956 | 3.56% | 48699 | 2.07% | 59901 | 2.54% | 43393 | 1.84% | 81598 | 3.46% | 34431 | 1.46% |
| Unknown | 102351 | 4.34% | 83956 | 3.56% | 102115 | 4.33% | 107681 | 4.57% | 107681 | 4.57% | 126170 | 5.35% | 102587 | 4.35% | 69806 | 2.96% | 76174 | 3.23% |
| White | 83956 | 3.56% | 79239 | 3.36% | 106832 | 4.53% | 110133 | 4.67% | 107539 | 4.56% | 107775 | 4.57% | 86550 | 3.67% | 57307 | 2.43% | 55420 | 2.35% |
| **Ethnicity (% of patients)** |  |  |  |  |  |  |  |  |  |  |  |  |  |  |  |  |  |  |
| Hispanic or Latino | 81362 | 3.45% | 73580 | 3.12% | 74287 | 3.15% | 76881 | 3.26% | 84192 | 3.57% | 110133 | 4.67% | 90795 | 3.85% | 99285 | 4.21% | 82541 | 3.50% |
| Non-Hispanic or Latino | 57779 | 2.45% | 34196 | 1.45% | 86786 | 3.68% | 83956 | 3.56% | 109426 | 4.64% | 125934 | 5.34% | 112020 | 4.75% | 81362 | 3.45% | 57779 | 2.45% |
| Unknown | 58015 | 2.46% | 58015 | 2.46% | 84192 | 3.57% | 109898 | 4.66% | 93154 | 3.95% | 152112 | 6.45% | 173336 | 7.35% | 126170 | 5.35% | 57779 | 2.45% |
| Patient Declined to Answer | 71929 | 3.05% | 83956 | 3.56% | 97399 | 4.13% | 102728 | 4.36% | 107539 | 4.56% | 107539 | 4.56% | 83956 | 3.56% | 57779 | 2.45% | 71693 | 3.04% |

*Note.* Recent clinical psychiatric diagnoses are defined as psychiatric diagnoses up to 3 years before the patient’s first COVID-19 reverse transcription-polymerase chain reaction (RT-PCR) test after March 2020.

# eTable 3 Prevalence of pre-COVID clinical psychiatric diagnoses.

|  | **With Recent Clinical Psychiatric Diagnoses (n=898,293)** | | | | | | | | |  | **Without Recent Clinical Psychiatric Diagnoses (n=1,460,025)** | | | | | | | | |
| --- | --- | --- | --- | --- | --- | --- | --- | --- | --- | --- | --- | --- | --- | --- | --- | --- | --- | --- | --- |
|  | **Monthly N** | | | | | **Monthly %** | | | |  | **Monthly N** | | | | | **Monthly %** | | | |
| Month | COVID-19 positive hospitalized patients | Anxiety Disorder | Mood Disorder | Psychosis | SUD | Anxiety Disorder | Mood Disorder | Psychosis | SUD | Month | COVID-19 hospitalization | Anxiety Disorder | Mood Disorder | Psychosis | SUD | Anxiety Disorder | Mood Disorder | Psychosis | SUD |
| Mar-20 | 5636 | 2847 | 2648 | 984 | 1084 | 50.5% | 47.0% | 17.5% | 19.2% | Mar-20 | 7271 | 1866 | 1983 | 1472 | 4341 | 25.7% | 27.3% | 20.2% | 59.7% |
| Apr-20 | 6979 | 2648 | 2437 | 1197 | 1152 | 37.9% | 34.9% | 17.2% | 16.5% | Apr-20 | 8502 | 2323 | 2394 | 1764 | 4561 | 27.3% | 28.2% | 20.7% | 53.6% |
| May-20 | 6479 | 3984 | 3444 | 1436 | 1193 | 61.5% | 53.2% | 22.2% | 18.4% | May-20 | 10695 | 2634 | 2293 | 1689 | 4851 | 24.6% | 21.4% | 15.8% | 45.4% |
| Jun-20 | 5583 | 3955 | 3435 | 1740 | 1967 | 70.8% | 61.5% | 31.2% | 35.2% | Jun-20 | 12426 | 2784 | 2207 | 1794 | 4975 | 22.4% | 17.8% | 14.4% | 40.0% |
| Jul-20 | 8190 | 4494 | 2645 | 1993 | 1835 | 54.9% | 32.3% | 24.3% | 22.4% | Jul-20 | 12850 | 3147 | 2353 | 1685 | 4983 | 24.5% | 18.3% | 13.1% | 38.8% |
| Aug-20 | 9102 | 4756 | 2536 | 2664 | 2543 | 52.3% | 27.9% | 29.3% | 27.9% | Aug-20 | 13734 | 4669 | 1963 | 2287 | 4752 | 34.0% | 14.3% | 16.7% | 34.6% |
| Sep-20 | 9744 | 3875 | 3855 | 2515 | 2987 | 39.8% | 39.6% | 25.8% | 30.7% | Sep-20 | 17543 | 1873 | 1736 | 1648 | 3163 | 10.7% | 9.9% | 9.4% | 18.0% |
| Oct-20 | 10008 | 2964 | 4179 | 2304 | 3874 | 29.6% | 41.8% | 23.0% | 38.7% | Oct-20 | 18274 | 1754 | 1687 | 1493 | 2848 | 9.6% | 9.2% | 8.2% | 15.6% |
| Nov-20 | 12641 | 2723 | 5322 | 2456 | 3494 | 21.5% | 42.1% | 19.4% | 27.6% | Nov-20 | 20121 | 1532 | 1502 | 1395 | 2975 | 7.6% | 7.5% | 6.9% | 14.8% |
| Dec-20 | 13747 | 2103 | 5492 | 2856 | 3361 | 15.3% | 39.9% | 20.8% | 24.4% | Dec-20 | 24891 | 1442 | 1334 | 1385 | 2564 | 5.8% | 5.4% | 5.6% | 10.3% |
| Jan-21 | 13958 | 1346 | 6226 | 3795 | 3864 | 9.6% | 44.6% | 27.2% | 27.7% | Jan-21 | 25430 | 1382 | 1163 | 1064 | 2281 | 5.4% | 4.6% | 4.2% | 9.0% |
| Feb-21 | 18145 | 1354 | 7463 | 3745 | 4284 | 7.5% | 41.1% | 20.6% | 23.6% | Feb-21 | 28623 | 1327 | 1040 | 861 | 2463 | 4.6% | 3.6% | 3.0% | 8.6% |
| Mar-21 | 18751 | 1293 | 3412 | 3975 | 2341 | 6.9% | 18.2% | 21.2% | 12.5% | Mar-21 | 28623 | 1453 | 2347 | 1548 | 3375 | 5.1% | 8.2% | 5.4% | 11.8% |
| Apr-21 | 24097 | 1093 | 2645 | 2961 | 2493 | 4.5% | 11.0% | 12.3% | 10.3% | Apr-21 | 28931 | 1586 | 2774 | 1954 | 3435 | 5.5% | 9.6% | 6.8% | 11.9% |
| May-21 | 24361 | 1232 | 1926 | 1974 | 1431 | 5.1% | 7.9% | 8.1% | 5.9% | May-21 | 32239 | 1679 | 2937 | 2371 | 3187 | 5.2% | 9.1% | 7.4% | 9.9% |
| Jun-21 | 23966 | 1238 | 1893 | 1648 | 1183 | 5.2% | 7.9% | 6.9% | 4.9% | Jun-21 | 32085 | 1794 | 3346 | 2176 | 2957 | 5.6% | 10.4% | 6.8% | 9.2% |
| Jul-21 | 26283 | 1367 | 1847 | 1436 | 894 | 5.2% | 7.0% | 5.5% | 3.4% | Jul-21 | 35278 | 1774 | 3422 | 2061 | 2756 | 5.0% | 9.7% | 5.8% | 7.8% |
| Aug-21 | 25678 | 1562 | 2514 | 1238 | 799 | 6.1% | 9.8% | 4.8% | 3.1% | Aug-21 | 27199 | 1403 | 3648 | 1726 | 2864 | 5.2% | 13.4% | 6.3% | 10.5% |
|  | COVID-19 positive nonhospitalized patients | Anxiety Disorder | Mood Disorder | Psychosis | SUD | Anxiety Disorder | Mood Disorder | Psychosis | SUD |  | COVID-19 positive nonhospitalized patients | Anxiety Disorder | Mood Disorder | Psychosis | SUD | Anxiety Disorder | Mood Disorder | Psychosis | SUD |
| Mar-20 | 4471 | 2457 | 1974 | 843 | 2084 | 55.0% | 44.2% | 18.9% | 46.6% | Mar-20 | 13048 | 1453 | 1668 | 1065 | 4764 | 11.1% | 12.8% | 8.2% | 36.5% |
| Apr-20 | 7054 | 2764 | 2645 | 1106 | 2187 | 39.2% | 37.5% | 15.7% | 31.0% | Apr-20 | 11825 | 1643 | 1783 | 1102 | 5753 | 13.9% | 15.1% | 9.3% | 48.7% |
| May-20 | 7178 | 3414 | 3548 | 2645 | 2227 | 47.6% | 49.4% | 36.8% | 31.0% | May-20 | 12488 | 1432 | 1536 | 1437 | 6704 | 11.5% | 12.3% | 11.5% | 53.7% |
| Jun-20 | 7824 | 3504 | 4585 | 2725 | 3164 | 44.8% | 58.6% | 34.8% | 40.4% | Jun-20 | 15902 | 1327 | 1665 | 1394 | 6344 | 8.3% | 10.5% | 8.8% | 39.9% |
| Jul-20 | 8544 | 4046 | 4292 | 2194 | 3119 | 47.4% | 50.2% | 25.7% | 36.5% | Jul-20 | 16514 | 1436 | 1682 | 1542 | 6143 | 8.7% | 10.2% | 9.3% | 37.2% |
| Aug-20 | 8867 | 4154 | 4338 | 2872 | 3794 | 46.8% | 48.9% | 32.4% | 42.8% | Aug-20 | 21458 | 1192 | 1340 | 1322 | 6745 | 5.6% | 6.2% | 6.2% | 31.4% |
| Sep-20 | 9339 | 1854 | 5472 | 2864 | 3725 | 19.9% | 58.6% | 30.7% | 39.9% | Sep-20 | 22121 | 1173 | 1297 | 1327 | 3425 | 5.3% | 5.9% | 6.0% | 15.5% |
| Oct-20 | 11624 | 1195 | 5026 | 2957 | 3296 | 10.3% | 43.2% | 25.4% | 28.4% | Oct-20 | 27218 | 1202 | 1304 | 1294 | 3547 | 4.4% | 4.8% | 4.8% | 13.0% |
| Nov-20 | 11823 | 1347 | 5342 | 3051 | 3085 | 11.4% | 45.2% | 25.8% | 26.1% | Nov-20 | 28900 | 1342 | 1044 | 1357 | 3364 | 4.6% | 3.6% | 4.7% | 11.6% |
| Dec-20 | 14033 | 1305 | 4921 | 2994 | 3353 | 9.3% | 35.1% | 21.3% | 23.9% | Dec-20 | 33997 | 1453 | 942 | 1192 | 3143 | 4.3% | 2.8% | 3.5% | 9.2% |
| Jan-21 | 14580 | 925 | 4246 | 2947 | 2847 | 6.3% | 29.1% | 20.2% | 19.5% | Jan-21 | 37412 | 1232 | 964 | 1244 | 3250 | 3.3% | 2.6% | 3.3% | 8.7% |
| Feb-21 | 15474 | 857 | 2924 | 2454 | 2647 | 5.5% | 18.9% | 15.9% | 17.1% | Feb-21 | 38125 | 1021 | 901 | 1282 | 3452 | 2.7% | 2.4% | 3.4% | 9.1% |
| Mar-21 | 15151 | 1062 | 2640 | 2273 | 1126 | 7.0% | 17.4% | 15.0% | 7.4% | Mar-21 | 37055 | 3136 | 1795 | 1467 | 3466 | 8.5% | 4.8% | 4.0% | 9.4% |
| Apr-21 | 18380 | 1153 | 2109 | 2394 | 1191 | 6.3% | 11.5% | 13.0% | 6.5% | Apr-21 | 38023 | 3472 | 2584 | 1948 | 3779 | 9.1% | 6.8% | 5.1% | 9.9% |
| May-21 | 19001 | 1264 | 1983 | 2416 | 1194 | 6.7% | 10.4% | 12.7% | 6.3% | May-21 | 43528 | 3395 | 2846 | 2940 | 3864 | 7.8% | 6.5% | 6.8% | 8.9% |
| Jun-21 | 24515 | 1573 | 1874 | 2436 | 1276 | 6.4% | 7.6% | 9.9% | 5.2% | Jun-21 | 36341 | 3845 | 3784 | 3834 | 3266 | 10.6% | 10.4% | 10.6% | 9.0% |
| Jul-21 | 24291 | 1094 | 1738 | 2582 | 983 | 4.5% | 7.2% | 10.6% | 4.0% | Jul-21 | 37870 | 4902 | 3986 | 3949 | 3547 | 12.9% | 10.5% | 10.4% | 9.4% |
| Aug-21 | 26228 | 839 | 1392 | 2741 | 809 | 3.2% | 5.3% | 10.5% | 3.1% | Aug-21 | 37870 | 4855 | 5383 | 4808 | 3613 | 12.8% | 14.2% | 12.7% | 9.5% |
|  | COVID-19 negative patients | Anxiety Disorder | Mood Disorder | Psychosis | SUD | Anxiety Disorder | Mood Disorder | Psychosis | SUD |  | COVID-19 negative patients | Anxiety Disorder | Mood Disorder | Psychosis | SUD | Anxiety Disorder | Mood Disorder | Psychosis | SUD |
| Mar-20 | 8427 | 2457 | 1936 | 849 | 3143 | 29.2% | 23.0% | 10.1% | 37.3% | Mar-20 | 12613 | 1783 | 986 | 1354 | 3764 | 14.1% | 7.8% | 10.7% | 29.8% |
| Apr-20 | 9046 | 3764 | 1864 | 1082 | 3017 | 41.6% | 20.6% | 12.0% | 33.4% | Apr-20 | 13179 | 1863 | 1093 | 1402 | 3654 | 14.1% | 8.3% | 10.6% | 27.7% |
| May-20 | 12795 | 3414 | 2163 | 1193 | 3264 | 26.7% | 16.9% | 9.3% | 25.5% | May-20 | 21550 | 2063 | 1193 | 1574 | 6781 | 9.6% | 5.5% | 7.3% | 31.5% |
| Jun-20 | 13762 | 4674 | 2756 | 1273 | 3364 | 34.0% | 20.0% | 9.3% | 24.4% | Jun-20 | 19853 | 1873 | 1303 | 1597 | 6504 | 9.4% | 6.6% | 8.0% | 32.8% |
| Jul-20 | 16351 | 4993 | 3545 | 1864 | 3342 | 30.5% | 21.7% | 11.4% | 20.4% | Jul-20 | 22794 | 1376 | 1434 | 1495 | 5952 | 6.0% | 6.3% | 6.6% | 26.1% |
| Aug-20 | 20178 | 4150 | 3583 | 2553 | 3403 | 20.6% | 17.8% | 12.7% | 16.9% | Aug-20 | 23303 | 1394 | 1536 | 1424 | 6813 | 6.0% | 6.6% | 6.1% | 29.2% |
| Sep-20 | 21841 | 3254 | 3634 | 2758 | 3232 | 14.9% | 16.6% | 12.6% | 14.8% | Sep-20 | 28959 | 1584 | 1754 | 1573 | 3754 | 5.5% | 6.1% | 5.4% | 13.0% |
| Oct-20 | 20217 | 2495 | 2946 | 2846 | 3585 | 12.3% | 14.6% | 14.1% | 17.7% | Oct-20 | 30204 | 1781 | 1896 | 1622 | 3604 | 5.9% | 6.3% | 5.4% | 11.9% |
| Nov-20 | 20101 | 2947 | 2547 | 2966 | 4109 | 14.7% | 12.7% | 14.8% | 20.4% | Nov-20 | 29582 | 1700 | 2135 | 1972 | 3767 | 5.7% | 7.2% | 6.7% | 12.7% |
| Dec-20 | 20642 | 1915 | 3228 | 3024 | 4253 | 9.3% | 15.6% | 14.6% | 20.6% | Dec-20 | 35238 | 1684 | 2545 | 1742 | 3864 | 4.8% | 7.2% | 4.9% | 11.0% |
| Jan-21 | 27368 | 1025 | 2325 | 3199 | 3849 | 3.7% | 8.5% | 11.7% | 14.1% | Jan-21 | 40950 | 1634 | 3547 | 1814 | 3974 | 4.0% | 8.7% | 4.4% | 9.7% |
| Feb-21 | 27523 | 1107 | 1848 | 3094 | 3124 | 4.0% | 6.7% | 11.2% | 11.4% | Feb-21 | 41629 | 1463 | 3784 | 1652 | 3974 | 3.5% | 9.1% | 4.0% | 9.5% |
| Mar-21 | 27716 | 1082 | 1648 | 2974 | 2642 | 3.9% | 5.9% | 10.7% | 9.5% | Mar-21 | 40385 | 3254 | 3984 | 3031 | 3964 | 8.1% | 9.9% | 7.5% | 9.8% |
| Apr-21 | 27948 | 1053 | 1747 | 1435 | 2332 | 3.8% | 6.3% | 5.1% | 8.3% | Apr-21 | 41629 | 3574 | 4123 | 3790 | 4109 | 8.6% | 9.9% | 9.1% | 9.9% |
| May-21 | 29726 | 1014 | 1434 | 1183 | 2134 | 3.4% | 4.8% | 4.0% | 7.2% | May-21 | 40498 | 3382 | 4784 | 3417 | 4205 | 8.4% | 11.8% | 8.4% | 10.4% |
| Jun-21 | 27562 | 920 | 1466 | 928 | 1792 | 3.3% | 5.3% | 3.4% | 6.5% | Jun-21 | 41460 | 3750 | 4591 | 3739 | 4402 | 9.0% | 11.1% | 9.0% | 10.6% |
| Jul-21 | 27910 | 945 | 1747 | 946 | 1702 | 3.4% | 6.3% | 3.4% | 6.1% | Jul-21 | 41516 | 4732 | 4894 | 4351 | 4561 | 11.4% | 11.8% | 10.5% | 11.0% |
| Aug-21 | 27446 | 839 | 1893 | 1446 | 1243 | 3.1% | 6.9% | 5.3% | 4.5% | Aug-21 | 40272 | 4431 | 4798 | 5473 | 4791 | 11.0% | 11.9% | 13.6% | 11.9% |

*Note.* Recent clinical psychiatric diagnoses are defined as psychiatric diagnoses up to 3 years before the patient’s first COVID-19 reverse transcription-polymerase chain reaction (RT-PCR) test after March 2020.

# eTable 4 Prevalence of pre-COVID clinical psychiatric diagnoses.

| **Clinical Psychiatric Disorders** | **2017** | | | **2018** | | | **2019** | | | **2020 (Jan-Mar)** | | |
| --- | --- | --- | --- | --- | --- | --- | --- | --- | --- | --- | --- | --- |
|  | n | % among Total Patients | % among Diagnosed Patients | n | % among Total Patients | % among Diagnosed Patients | n | % among Total Patients | % among Diagnosed Patients | n | % among Total Patients | % among Diagnosed Patients |
| Total Patients | 647594 | 27.46% |  | 621417 | 26.35% |  | 694053 | 29.43% |  | 395254 | 16.76% | 16.76% |
| Diagnosed | 225579 | 34.83% |  | 279484 | 44.98% |  | 324164 | 46.71% |  | 295240 | 74.70% |  |
| Anxiety Disorders | 52057 | 8.04% | 23.08% | 59508 | 9.58% | 21.29% | 68699 | 9.90% | 21.19% | 64827 | 16.40% | 21.96% |
| Mood Disorders | 67192 | 10.38% | 29.79% | 63503 | 10.22% | 22.72% | 85552 | 12.33% | 26.39% | 74251 | 18.79% | 25.15% |
| Psychosis | 43860 | 6.77% | 19.44% | 57575 | 9.27% | 20.60% | 58032 | 8.36% | 17.90% | 68734 | 17.39% | 23.28% |
| Substance Use Disorders | 62469 | 9.65% | 27.69% | 98898 | 15.91% | 35.39% | 111882 | 16.12% | 34.51% | 87428 | 22.12% | 29.61% |

# eFigure 1 Geographic distributions of health facilities in Healthix.


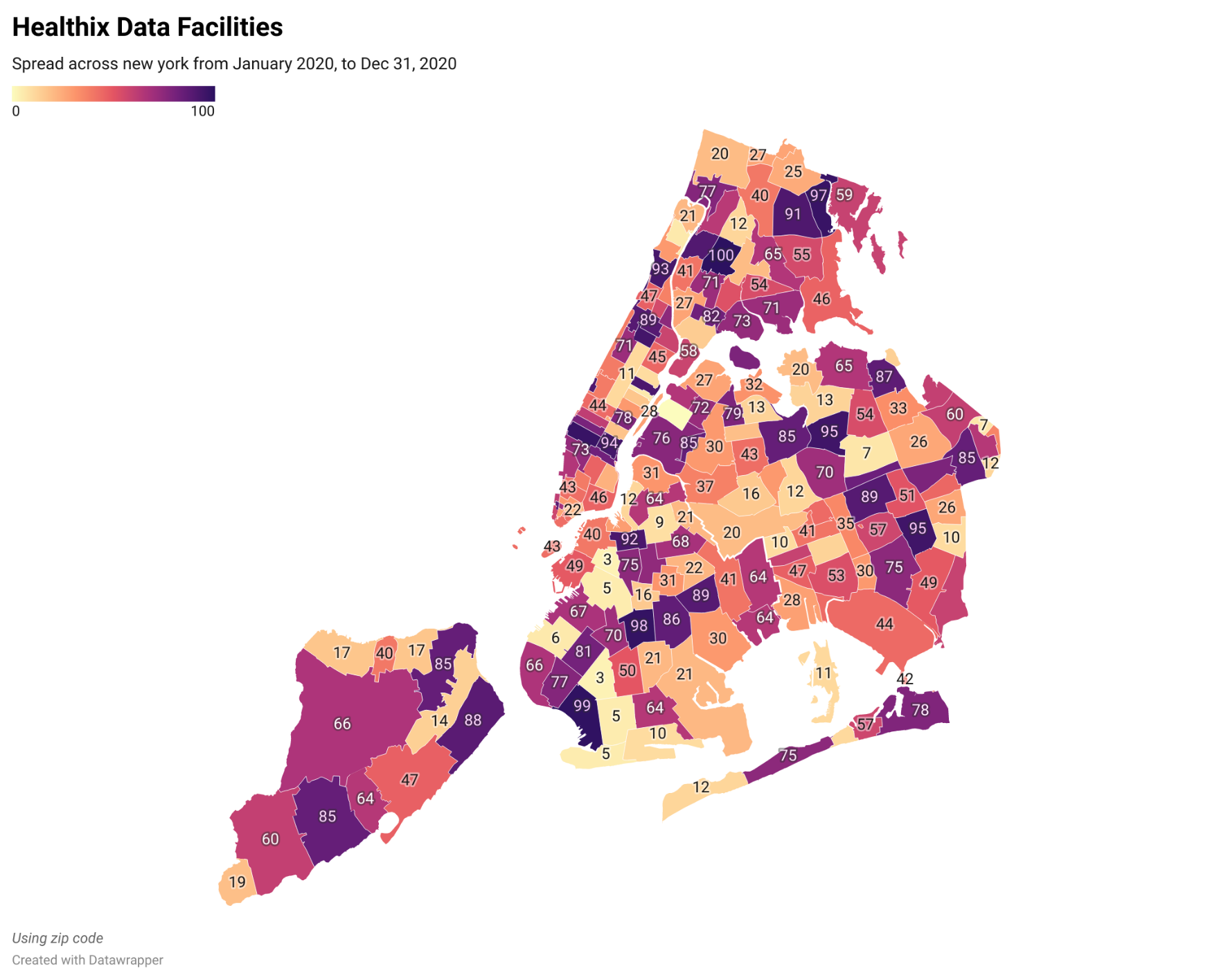

Supplement: Supplementary file 1 — Supplementary Materials [file 41398_2022_2255_MOESM1_ESM.docx]
